# Role of cattle treated with deltamethrine in areas with a high population of *Anopheles arabiensis *in Moshi, Northern Tanzania

**DOI:** 10.1186/1475-2875-6-109

**Published:** 2007-08-08

**Authors:** Aneth M Mahande, Franklin W Mosha, Johnson M Mahande, Eliningaya J Kweka

**Affiliations:** 1 KCM College of Tumaini University, PO Box 2240, Moshi, Tanzania; 2 Ifakara Health Research and Development Centre, Department of Public Health Entomology, PO Box 53, Ifakara-Morogoro, Tanzania; 3 Kilimanjaro Christian Medical Centre, PO Box 3010, Moshi, Tanzania

## Abstract

**Background:**

Malaria control measures were initiated from in October 2005 to August 2006 in the Lower Moshi irrigation schemes, Tanzania. This manuscript reports on the entomological evaluation of the impact of pyrethroid-treated cattle in reducing the population of the *Anopheles arabiensis *for selected houses in the Lower Moshi irrigation scheme.

**Methods:**

Cattle were sprayed with the pyrethroid (deltamethrin) acaricide. Grazing and non-grazing cattles were compared and assessed for difference in knockdown resistance (kdr) time using cone or contact bioassay and residual effect (mortality). In experimental huts, mortality was compared between the huts with treated and untreated cattle.

**Results:**

Results from contact bioassays of cattle treated with deltamethrin showed a knockdown effect of 50% within 21 days for grazing cattle and 29 days for non-grazing cattle. Residual effect at 50% was achieved within 17 days for grazing cattle compared to 24 days for inshed cattle. In discussing the results, reference has been made to the exophilic and zoophilic tendencies of *An. arabiensis*, which are conducive for zooprophylaxis.

Experimental studies in Verandah huts at Mabogini compared *An. arabiensis *and *Culex spp *collected from huts with different baits, i e. human, untreated cow and treated cow. Results indicate higher mortality rates in mosquitoes collected from the hut containing the treated cow (mean = 2) compared to huts with untreated cow (mean = 0.3) and human (mean = 0.8). A significantly higher number of Culex *spp*. was recorded in huts with treated cows compared to the rest.

**Conclusion:**

This study has demonstrated the role of cattle treated with pyrethroid in the control of malaria and reduction of vector density. It showed that, in areas with a predominant *An. arabiensis *population, cattle should be placed close to dwelling houses in order to maximize the effects of zooprophylaxis. Protective effects of cattle can further be enhanced by regular treatment with pyrethroids at least every three weeks. This paper demonstrates that cattle can be considered as Insecticide-Treated Material (ITM) as long as acaricide treatment is conducted regularly.

## Background

The greatest impact of malaria in terms of morbidity and mortality is in sub-Saharan African, where *Plasmodium falciparum *is predominant, accounting for more than 25% of childhood mortality outside the neonatal period[1]. Recent reports on disease burden of malaria have revealed that about one million deaths occur yearly in Africa from the direct effects of malaria. Of these, more than 75% occurs in children[2]. The population at risk continues to be significant (nearly 300–515 million clinical cases in the world). Malaria's patchy nature combined with technical problems such as drug resistance, complex vector ecologies and strong socio-cultural perspectives provide a challenge to public health authorities [3]. In many areas, malaria has been associated with environmental conditions, including land and water management [4]. It is estimated that 90% of the global burden of the disease is attributable to environmental factors [5].

Current mosquito control methods rely heavily on the use of insecticides through larviciding, residual house spraying, insecticide-treated nets and other personal protection methods [6,7]. This widespread use of insecticides has lead to the development of many insecticide-resistant mosquito populations, thus leading to failure of malaria disease control. Because of the insecticide resistance in mosquitoes and the concern about environmental pollution when using pesticides, there has been an increased emphasis on the development of alternative mosquito control technologies [8,9].

There is, therefore, an urgent need to look at novel techniques, which will complement the existing strategies; one of such method is the use of zooprophylaxis in areas where large numbers of livestock exist and where is the predominant vector of *Anopheles arabiensis *[10]

In this study the effect of deltamethrine applied on cattle, on the target mosquito species and on natural mosquito species (in experimental huts) were thoroughly assessed. The acaricide used was a deltamethrin formulation, commonly used to control tsetse and ticks in the study area. This study focused on two aspects in particular: (i) the knockdown and residual effect of *An. arabiensis *on treated cow by using contact or cone bioassay, (ii) The behavioural aspect of mosquitoes on treated cows as compared to mosquitoes on humans and untreated cows.

The study observed that in-shade treated animals have higher protection than treated grazing animals. This study assessed the impact of insecticide-treated cattle with pyrethroids in reducing the population of the *An. arabiensis*, by means of killing rather than simply diverting host-seeking mosquitoes in areas with a high population of *An. arabiensis*.

## Materials and methods

### Study site

This study was carried out in Lower Moshi area, Kilimanjaro region, Northern Tanzania. The average population per village was 2,842[11,12]. Lower Moshi area is at altitude of about 800 m above sea level. Rainfall is seasonally concentrated in March–May accounting for about 60% of the annual total of 800 mm precipitation at Moshi Town (10–15 km north of the study area) while the remainder falls during October–December. Between these two rainy seasons are hot dry seasons during January–February and a cool dry season during June–September.

### Effect of cattle treated with pyrethroid acaricide on *An. arabiensis*

#### Contact or cone bioassay

A total of eight cows were selected, half of them were left untreated (control) and the rest were treated with deltamethrin, the normal practice of cattle protection against ticks and tsetse flies. Of the four cattle in each group, two were kept under shelter at all times (in-shed), while the other two were grazed outdoors during the day as per recommendation of Hewitt and Rowland [13].

To avoid possible insecticide contamination, the experimental sites for untreated and treated cattle were approximately one km from each other and during the day time they were grazed separately. Residual insecticide on the animals was carried out by contact bioassay method as per WHO guidelines [14].

Five unfed, 2–5 days old *An. arabiensis *mosquitoes were exposed on the treated and untreated animals (control) for three minutes and then transferred into a clean paper cup containing a piece of cotton soaked in 10% sugar solution as food source. Immediate (KDR) and delayed mortality rates were recorded after one hour and 24 hours respectively.

#### Experimental hut trials

The effect of cattle treated with pyrethroids on *An. arabiensis *was also assessed in the experimental huts as follows: the treated cow, human and untreated cow were rotated according to a latin square design in three experimental huts in order to minimize the influencing factors such as variation in wind direction, collector ability and mosquitoes relative abundance. One cow was treated with deltamethrin according to the community normal practice and as per recommendations of the manufacturer (i.e. 1 ml of deltamethrin mixed with one liter of water, 2.5 liters were used to spray one cow). Before sunset, the test calves (one year old) were tethered, one in each hut and volunteer sleeper in another hut. All verandahs were left open in the evening at 6.00 pm to allow mosquitoes to enter into the hut. The verandahs were closed in the morning at 5.00 a.m in order to prevent mosquitoes from escaping. Mosquitoes were collected from the verandah and inside the hut using an aspirator. Collected mosquitoes were sorted and recorded according to their species [15] and abdominal condition (unfed or fed). Live mosquitoes were transferred into paper cups and provided with sugar solution and held for 24 hrs.

The percentage of blood-fed, repellence and mortality were calculated according to the following formulae:-

a) Percentage Blood fed=fed+semigravidTotal mosquitoes collected×100
 MathType@MTEF@5@5@+=feaafiart1ev1aaatCvAUfKttLearuWrP9MDH5MBPbIqV92AaeXatLxBI9gBaebbnrfifHhDYfgasaacH8akY=wiFfYdH8Gipec8Eeeu0xXdbba9frFj0=OqFfea0dXdd9vqai=hGuQ8kuc9pgc9s8qqaq=dirpe0xb9q8qiLsFr0=vr0=vr0dc8meaabaqaciaacaGaaeqabaqabeGadaaakeaacqqGHbqycqqGPaqkcqqGGaaicqqGqbaucqqGLbqzcqqGYbGCcqqGJbWycqqGLbqzcqqGUbGBcqqG0baDcqqGHbqycqqGNbWzcqqGLbqzcqqGGaaicqqGcbGqcqqGSbaBcqqGVbWBcqqGVbWBcqqGKbazcqqGGaaicqqGMbGzcqqGLbqzcqqGKbazcqGH9aqpdaWcaaqaaiabbAgaMjabbwgaLjabbsgaKjabgUcaRiabbohaZjabbwgaLjabb2gaTjabbMgaPjabbEgaNjabbkhaYjabbggaHjabbAha2jabbMgaPjabbsgaKbqaaiabbsfaujabb+gaVjabbsha0jabbggaHjabbYgaSjabbccaGiabb2gaTjabb+gaVjabbohaZjabbghaXjabbwha1jabbMgaPjabbsha0jabb+gaVjabbwgaLjabbohaZjabbccaGiabbogaJjabb+gaVjabbYgaSjabbYgaSjabbwgaLjabbogaJjabbsha0jabbwgaLjabbsgaKbaacqGHxdaTcqaIXaqmcqaIWaamcqaIWaamaaa@8393@

b) Percentage Repellence=mosquitoes in verandah+in window trapTotal mosquitoes collected×100
 MathType@MTEF@5@5@+=feaafiart1ev1aaatCvAUfKttLearuWrP9MDH5MBPbIqV92AaeXatLxBI9gBaebbnrfifHhDYfgasaacH8akY=wiFfYdH8Gipec8Eeeu0xXdbba9frFj0=OqFfea0dXdd9vqai=hGuQ8kuc9pgc9s8qqaq=dirpe0xb9q8qiLsFr0=vr0=vr0dc8meaabaqaciaacaGaaeqabaqabeGadaaakeaacqqGIbGycqqGPaqkcqqGGaaicqqGqbaucqqGLbqzcqqGYbGCcqqGJbWycqqGLbqzcqqGUbGBcqqG0baDcqqGHbqycqqGNbWzcqqGLbqzcqqGGaaicqqGsbGucqqGLbqzcqqGWbaCcqqGLbqzcqqGSbaBcqqGSbaBcqqGLbqzcqqGUbGBcqqGJbWycqqGLbqzcqGH9aqpdaWcaaqaaiabb2gaTjabb+gaVjabbohaZjabbghaXjabbwha1jabbMgaPjabbsha0jabb+gaVjabbwgaLjabbohaZjabbccaGiabbMgaPjabb6gaUjabbccaGiabbAha2jabbwgaLjabbkhaYjabbggaHjabb6gaUjabbsgaKjabbggaHjabbIgaOjabgUcaRiabbMgaPjabb6gaUjabbccaGiabbEha3jabbMgaPjabb6gaUjabbsgaKjabb+gaVjabbEha3jabbccaGiabbsha0jabbkhaYjabbggaHjabbchaWbqaaiabbsfaujabb+gaVjabbsha0jabbggaHjabbYgaSjabbccaGiabb2gaTjabb+gaVjabbohaZjabbghaXjabbwha1jabbMgaPjabbsha0jabb+gaVjabbwgaLjabbohaZjabbccaGiabbogaJjabb+gaVjabbYgaSjabbYgaSjabbwgaLjabbogaJjabbsha0jabbwgaLjabbsgaKbaacqGHxdaTcqaIXaqmcqaIWaamcqaIWaamaaa@A347@

c) Percentage mortality=Dead (immediate and delayed)Total mosquitoes collected×100
 MathType@MTEF@5@5@+=feaafiart1ev1aaatCvAUfKttLearuWrP9MDH5MBPbIqV92AaeXatLxBI9gBaebbnrfifHhDYfgasaacH8akY=wiFfYdH8Gipec8Eeeu0xXdbba9frFj0=OqFfea0dXdd9vqai=hGuQ8kuc9pgc9s8qqaq=dirpe0xb9q8qiLsFr0=vr0=vr0dc8meaabaqaciaacaGaaeqabaqabeGadaaakeaacqqGJbWycqqGPaqkcqqGGaaicqqGqbaucqqGLbqzcqqGYbGCcqqGJbWycqqGLbqzcqqGUbGBcqqG0baDcqqGHbqycqqGNbWzcqqGLbqzcqqGGaaicqqGTbqBcqqGVbWBcqqGYbGCcqqG0baDcqqGHbqycqqGSbaBcqqGPbqAcqqG0baDcqqG5bqEcqGH9aqpdaWcaaqaaiabbseaejabbwgaLjabbggaHjabbsgaKjabbccaGiabcIcaOiabbMgaPjabb2gaTjabb2gaTjabbwgaLjabbsgaKjabbMgaPjabbggaHjabbsha0jabbwgaLjabbccaGiabbggaHjabb6gaUjabbsgaKjabbccaGiabbsgaKjabbwgaLjabbYgaSjabbggaHjabbMha5jabbwgaLjabbsgaKjabcMcaPaqaaiabbsfaujabb+gaVjabbsha0jabbggaHjabbYgaSjabbccaGiabb2gaTjabb+gaVjabbohaZjabbghaXjabbwha1jabbMgaPjabbsha0jabb+gaVjabbwgaLjabbohaZjabbccaGiabbogaJjabb+gaVjabbYgaSjabbYgaSjabbwgaLjabbogaJjabbsha0jabbwgaLjabbsgaKbaacqGHxdaTcqaIXaqmcqaIWaamcqaIWaamaaa@94EA@

### Data analysis

The data entry was done in Microsoft Excel (2000) and analysis was carried out using statistical package for social science (SPSS) version 10 program. The significance test was estimated assuming an α (two sided) = 0.05). Other data were analysed by using EpiInfo™ Version 3.2.2 programme where χ^2 ^and P value were calculated.

### Ethical considerations

Before conducting this study, ethical clearance was sought from the Kilimanjaro Christian Medical College Research Ethics Committee. Permission from the district and respective village authorities was obtained also. Both verbal and written informed consent was obtained from the head of the household of the respective households that were selected for the study. Antimalaria drugs (sulphadoxine/pyrimethamine, SP) were kept at the field station for emergency purposes, although luckily during the course of the experiment none of the sleepers contacted malaria. Among households where field experiments were conducted, the mosquito density was reduced by the pyrethrum-spraying catches. Customs and norms of the respective community at the study area were maintained and respected.

## Results

### Effect of cattle treated with pyrethroids acaricide on *An. arabiensis*

**Figure 1:**
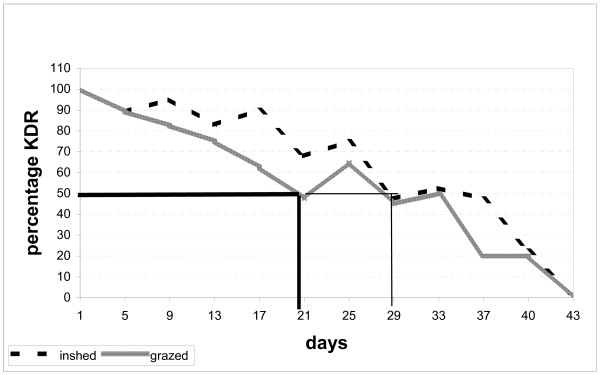
Knockdown effect of acaricide (applied on cows) on *An. arabiensis*.

**Figure 2:**
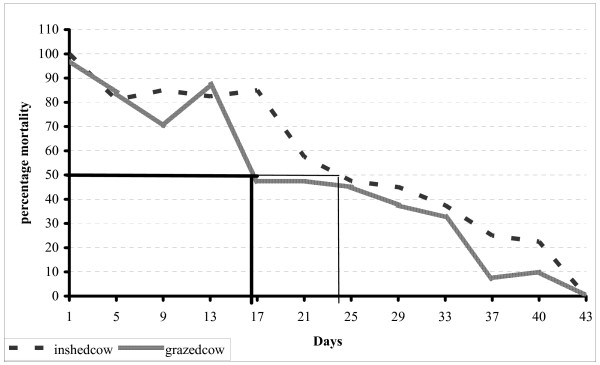
Residual effect of acaricide (applied on cows) on *An. arabiensis *mortality.

The effect of cattle treated with pyrethroids on mosquitoes was investigated in the field using cone bioassay. Knock down resistance (KDR) and mortality (residual effect) for in-shed and grazing cows were recorded and summarized as shown in Figures 1 and 2. A total of 948 female *An. arabiensis *mosquitoes were tested on treated cows. The insecticide was found to be effective for almost a month giving more than 50% knockdown at three weeks post-treatment for grazing animals, and four weeks for in-shed animals (Figure 1). Also the acaricide achieved more than 50% mortality of mosquitoes held for over 24 hours up to two weeks for grazing cows and three weeks for in-shed cows (Figure 2)

The chi-square and P value for KDR and mortality rate of mosquitoes on the treated cows were calculated (Table 1). Both KDR and mortality rate of mosquito were associated with duration post-treatment. The chi-square in knockdown mosquito for in-shed and grazing animal were (χ^2 ^= 8.9, P = 0.003), and (χ^2 ^= 9.8, P = 0.002), respectively. Similarly, the chi-square in 24 hrs mortality rate for in-shed cows was (χ^2 ^= 10.2, P = 0.001), while that of grazing animal it was (χ^2 ^= 9.99, P = 0.002).

### Experimental hut studies

Higher mortality rates were observed in mosquitoes collected from the experimental hut containing treated cows (mean = 2) compared to untreated cows (mean = 0.3) and humans (mean = 0.8). However, no statistical significant difference was found in mortality in the hut with treated cows and that with untreated cows (P > 0.05). Significantly higher *Culex spp*. mortalities were recorded in the experimental huts with treated cows compared with the rest.

## Discussion

The results from field and experimental huts have demonstrated that cattle treated with the acaricide deltamethrin can offer protection of humans against *An. arabiensis*, both through repellency and mortality. The same observation had previously been made by others [16-19]. The results suggested that in order to achieve 50% knockdown (immediate mortality), *An. arabiensis *re-treatment must be done after 21 days for grazing cattle and 29 days for in-shed cattle. In order to achieve 50% mortality (delayed mortality due to residual effect of acaricide), retreatment must be done before 17 days for grazing cattle and 25 days for in-shed cattle [20]. The effect of pyrethroids applied on cattle and, in particular, deltamethrin in killing mosquitoes landing on the cattle has also been demonstrated elsewhere [13,16,21-23]. The use of insecticide-treated cattle can cause the reduction of *An. arabiensis *population because this insect has an overlapping distribution [15,24-27] hence reducing the malaria transmission in community.

Result from the experimental huts where untreated cows, acaricide-treated cows with and humans were rotated also indicates a slightly higher performance of the treated cows particularly in terms of mortality rates on *An. arabiensis *and *Culex spp*, as had been described elsewhere [13,16,21,22,28]

## Conclusion

The present studies have shed some more light on the positive impact of pyrethroid-treated cattle in reducing man-vector contact. Therefore, malaria transmission in areas with a predominant *An. arabiensis *mosquito population can be reduced by zooprophylaxis. Cattle treated with pyrethroid acaricides will not simply divert host-seeking mosquitoes from man, but will also cause mortality of more than half of them. In this way, the negative impact of zooprophylaxis, whereby mosquito densities may increase due the presence of a readily available blood meal source, can be minimized. Cattle also serve as dead-end hosts since malaria parasites cannot develop in their red blood cells.

In pastoral communities, where dwelling areas are shared with cattle, routine application of acaricides on the animals for tick and tsetse control may also serve as an alternative method to Insecticide-Treated Materials (ITMs).

## Competing interests

The author(s) declare that they have no competing interests.

## Authors' contributions

AMM and FWM conceived and designed the study, participated in analysis and interpretation of data and contributed to the drafting of the manuscript. JMM carried out the analyses of mosquitoes assisted with data analysis and interpretation and were involved in the drafting of the manuscript. EJK designed and coordinated the study, participated in the analysis and interpretation of results, and was involved it the drafting of the manuscript and critical evaluation thereof. All authors read and approved the manuscript.

**Table 1:** Mortality rates of *An. arabiensis *collected from the experimental huts.

**Paired sample**	**No. of dead**	**Mean**	**SD**	**SE**	**95% CI**	**T-test**	**P-value**
1^st ^paired sample							
Untreated cow-	2	0.33	0.8	0.3	-4.68–1.35	-1.42	0.22
Treated cow	56	2.0	2.6	1.06			
2^nd ^paired sample							
Untreated cow-	2	0.33	0.82	0.33	-2.57–1.57	-0.6	0.56
Human	5	0.83	1.6	0.6			
3^rd ^paired sample							
Treated cow-	56	2.00	2.6	1.06	-0.06–2.39	2.5	0.06
Human	5	0.83	1.6	0.65			

## References

[B1] Winstanley P, Ward S, Snow R, Breckenridge A (2004). Therapy of falciparum malaria in Sub-saharan Africa: from molecule to policy. Clin Microbiol Rev.

[B2] Snow RW, Craig M, Deichmann U, Marsh K (1999). Estimating mortality, morbidity and disability due to malaria among Africa's non-pregnant population. Bull World Health Organ.

[B3] Greenwood B, Mutabingwa T (2002). Malaria in 2002. Nature.

[B4] Mutero CM, Kabutha C, Kimani V, Kabuage L, Gitau G, Ssennyonga J, Githure J, Muthami L, Kaida A, Musyoka L, Kiarie E, Oganda M (2004). A transdisciplinary perspective on the links between malaria and agroecosystems in Kenya. Acta Trop.

[B5] Hay S, Guerra C, Tatem A, Noor A, Snow R (2004). The global distribution and population at risk of malaria: past, present, and future. Lancet Infect Dis.

[B6] Magesa SM, Lengeler C, deSavigny D, Miller JE, Njau RAJ, Kramer K, Kitua A, Mwita A (2005). Creating an "enabling environment" for taking insecticide treated nets to national scale: the Tanzanian experience. Malar J.

[B7] Kuntz KJ, Olson JK, Rade DJ (1982). Role of domestic animals as hosts for blood-seeking females of *Plasmodium columbiae *and other mosquito species in Texas rice fields. Mosq News.

[B8] Kline DL (1994). Olfactory attractants for mosquito surveillance and control: 1-octen-3-ol. J Am Mosq Control Assoc.

[B9] Kline DL (1998). Olfactory responses and field attraction of mosquitoes to volatiles from Limburger cheese and human foot odor. J Vector Ecol.

[B10] Ijumba JN, Mwangi RW, Beier JC (1990). Malaria transmission potential of Anopheles mosquitoes in the Mwea-Tebere irrigation scheme, Kenya. Med Vet Entomol.

[B11] National Bureau of Statistics, ORC Macro (2005). Tanzania Demographic and Health Survey 2004–5: Key findings.

[B12] National census report 2002. http://www.tanzania.go.tz/censusdb/index.html.

[B13] Hewitt S, Rowland M (1999). Control of zoophylic malaria vectors by applying pyrethroid insecticides to cattle. Trop Med Int Health.

[B14] WHO (1996). Report of the WHO Informal Consultation on the Evaluation and testing of Insecticides.

[B15] Gillies TM, Coetzee M (1987). Supplement of the Anopheles of Africa South of Sahara (Afrotropical Region).

[B16] McLaughlin RE, Focks DA, Dame DA (1989). Residual activity of permethrin on cattle as determined by mosquito bioassays. J Am Mosq Contr Assoc.

[B17] Ralisoa R, Coluzzi M (1987). Genetical investigation of zoophilic and exophilic *An. arabiensis *from Antananarivo area (Madagascar). Parassitologia.

[B18] Schultz GW (1989). Animal influence on man-biting rates at a malarious site in Palawan, Philippines. Southeast Asian J Trop Med Public Health.

[B19] Hewitt S, Kamal M, Muhammad N, Rowland M (1994). An entomological investigation of the likely impact of cattle ownership on malaria in an Afghan refugee camp in the North West Frontier Province of Pakistan. Med Vet Entomol.

[B20] Nasci RS, McLaughlin RE, Focks D, Billodeaux J (1990). Effects of topically treating cattle with permethrin on *Psorophora columbiae *(Diptera: Culicidae) blood feeding in a Southwest Louisiana rice-pasture ecosystem. J Econ Entomol.

[B21] Habtewold T, Prior A, Torr JS, Gibson G (2004). Could Insecticide-treated cattle reduce Afrotropical malaria transmission? Effects of deltamethrin-treated zebu on *Anopheles arabiensis *behaviour and survival in Ethiopia. Med Vet Entomol.

[B22] Habtewold T, Walker AR, Curtis CF, Osir EO, Thapa N (2001). The feeding behaviour and Plasmodium infection of Anopheles mosquitoes in southern Ethiopia in relation to use of insecticide-treated livestock for malaria control. Trans R Soc Trop Med Hyg.

[B23] Hagmann R, Charlwood JD, Gil V, Ferreira C, do Rosario V, Smith TA (2003). Malaria and its possible control on the island of Principe. Malar J.

[B24] Ford J, Katondo KM (1973). Maps of tsetse flies (Glossina) distribution in Africa according to subgeneric groups on scale of 1:5000000. Bull Anim Health Prod Afr.

[B25] Saul A (2003). Zooprophylaxis or zoopotentiation: the outcome of introducing animals on vector transmission is highly dependent on the mosquito mortality while searching. Malar J.

[B26] Seyoum A, Balcha F, Balkew M, Ali A, Gebre-Michael T (2002). Impact of cattle keeping on human biting rate of Anopheline mosquitoes and malaria transmission around Ziway, Ethiopia. E Afr Med J.

[B27] Zebra E (1998). Insecticidal activity of pyrethroids on insects of medical importance. Parasitol Today.

[B28] Killeen GF, Smith TA (2007). Exploring the contributions of bed nets, cattle, insecticides and excito repellency to malaria control: a deterministic model of mosquito host-seeking behaviour and mortality. Trans R Soc Trop Med Hyg.

